# 10,11,12,13-Tetrahydro-4,5,9,14-tetra­azadibenz[*a*,*c*]anthracene–benzene-1,4-dicarboxylic acid (2/1)

**DOI:** 10.1107/S1600536808027785

**Published:** 2008-09-06

**Authors:** Chun-Bo Liu, Jian Wang, Xiu-Ying Li, Guang-Bo Che, Yang Liu

**Affiliations:** a Department of Chemistry, Jilin Normal University, Siping 136000, People’s Republic of China

## Abstract

In the title adduct, 2C_18_H_14_N_4_·C_8_H_6_O_4_, the centrosymmetric 1,4-benzene­dicarboxylic acid mol­ecule makes two O—H⋯·N hydrogen bonds to adjacent 10,11,12,13-tetra­hydro-4,5,9,14-tetra­azadibenzo[*a*,*c*]anthracene (TTBT) mol­ecules. Aromatic π–π stacking inter­actions occur between TTBT rings [centroid–centroid distance = 3.570 (3) Å], leading to a two-dimensional supra­molecular structure in the crystal.

## Related literature

For related literature, see: Che *et al.* (2006 ▶, 2008 ▶); Stephenson & Hardie (2006 ▶); Xu *et al.* (2008 ▶); Yao *et al.* (2008 ▶).
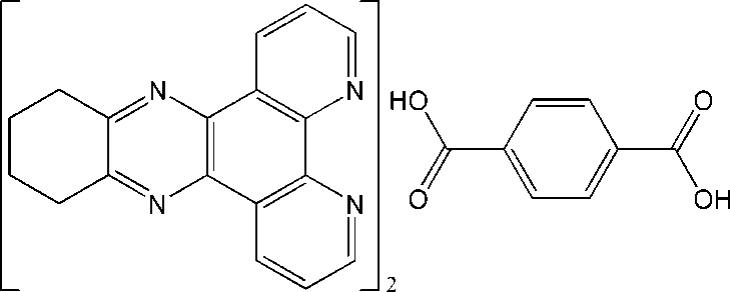


## Experimental

### 

#### Crystal data

2C_18_H_14_N_4_·C_8_H_6_O_4_
*M* *_r_* = 738.80
Triclinic, 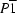

*a* = 7.266 (4) Å
*b* = 9.917 (5) Å
*c* = 13.564 (9) Å
α = 101.469 (9)°
β = 97.429 (9)°
γ = 109.697 (6)°
*V* = 881.2 (9) Å^3^
*Z* = 1
Mo *K*α radiation
μ = 0.09 mm^−1^
*T* = 292 (2) K
0.32 × 0.21 × 0.08 mm

#### Data collection

Bruker SMART CCD diffractometer
Absorption correction: multi-scan (*SADABS*; Bruker, 2002 ▶) *T*_min_ = 0.978, *T*_max_ = 0.992
7636 measured reflections
3444 independent reflections
1394 reflections with *I* > 2σ(*I*)
*R*_int_ = 0.047

#### Refinement

*R*[*F* ^2^ > 2σ(*F* ^2^)] = 0.058
*wR*(*F* ^2^) = 0.180
*S* = 0.93
3444 reflections
253 parameters
H-atom parameters constrained
Δρ_max_ = 0.34 e Å^−3^
Δρ_min_ = −0.18 e Å^−3^

### 

Data collection: *SMART* (Bruker, 2002 ▶); cell refinement: *SAINT* (Bruker, 2002 ▶); data reduction: *SAINT*; program(s) used to solve structure: *SHELXS97* (Sheldrick, 2008 ▶); program(s) used to refine structure: *SHELXL97* (Sheldrick, 2008 ▶); molecular graphics: *SHELXTL* (Sheldrick, 2008 ▶); software used to prepare material for publication: *SHELXTL*.

## Supplementary Material

Crystal structure: contains datablocks global, I. DOI: 10.1107/S1600536808027785/hb2788sup1.cif

Structure factors: contains datablocks I. DOI: 10.1107/S1600536808027785/hb2788Isup2.hkl

Additional supplementary materials:  crystallographic information; 3D view; checkCIF report

## Figures and Tables

**Table 1:** Hydrogen-bond geometry (Å, °)

*D*—H⋯*A*	*D*—H	H⋯*A*	*D*⋯*A*	*D*—H⋯*A*
O2—H2*A*⋯N2	0.82	2.02	2.694 (4)	139

**Fig. 1.**
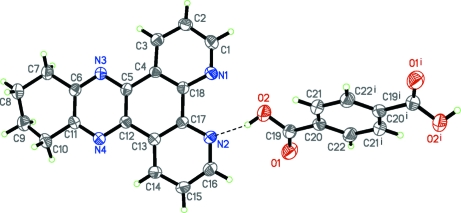
View of the molecular structure of (I). Displacement ellipsoids are drawn at the 30% probability level (arbitrary spheres for the H atoms). [Symmetry code: (i) 1 - x, -y, 3 - z.]

**Fig. 2.**
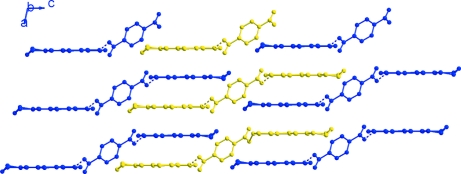
Packing diagram of the two-dimensional supramolecular structure of (I) formed viaπ–π interactions and hydrogen bonds. H atoms have been omitted.

## supplementary crystallographic information

### Comment

Current crystal engineering on the basis of the supramolecular architectures assembled from various noncovalent interactions, such as hydrogen bonds and π-π stacking interactions have been extensively studied owing to their novel topologies and potential applications as functional materials (Stephenson & Hardie, 2006; Yao *et al.*,2008). 1,10-Phenanthroline (phen) and its derivatives have been widely used to build novel supramolecular architectures (Xu, Li *et al.*, 2008; Che, Liu *et al.*, 2008). As a continuation of our studies in this area, we have prepared the title compound, (I), using the phen derivative 10,11,12,13-tetrahydro-4,5,9,14-tetraazadibenz[*a*,*c*]anthracene (TTBA).

The asymmetric unit of (I) consists of one TTBA molecule and half of a centrosymmetric 1,4-benzenedicarboxylic acid molecule (Fig. 1). The two components of (I) interact by way of O—H···N hydrogen bonds (Table 1). Furthermore, there are π-π aromatic stacking interactions involving TTBA ligands of adjacent units [centroid-centroid distance = 3.570 (3)Å], forming an intriguing two-dimensional supramolecular motif (Fig. 2).

### Experimental

The TTBA was synthesized according to the literature method (Che, Li *et al.*, 2006). TTBA (1.0 mmol) and 1,4-benzenedicarboxylic acid (0.5 mmol) were dissolved in aqueous solution and the mixture was sealed in a Teflon-lined autoclave and heated to 433 K for 4 d. Upon cooling and opening the bomb, colorless blocks of (I) were obtained.

### Refinement

The hydrogen atoms were positioned geometrically (C—H = 0.93 Å, O—H = 0.82Å) and refined as riding, with *U*_iso_(H)= 1.2*U*_eq_(carrier).

### Crystal data

**Table:**

2C_18_H_14_N_4_·C_8_H_6_O_4_	*Z* = 1
*M**_r_* = 738.80	*F*(000) = 386
Triclinic, *P*1	*D*_x_ = 1.392 Mg m^−^^3^
Hall symbol: -P 1	Mo *K*α radiation, λ = 0.71073 Å
*a* = 7.266 (4) Å	Cell parameters from 2386 reflections
*b* = 9.917 (5) Å	θ = 2.3–26.0°
*c* = 13.564 (9) Å	µ = 0.09 mm^−^^1^
α = 101.469 (9)°	*T* = 292 K
β = 97.429 (9)°	Block, colorless
γ = 109.697 (6)°	0.32 × 0.21 × 0.08 mm
*V* = 881.2 (9) Å^3^	

### Data collection

**Table:**

Bruker SMART CCD diffractometer	3444 independent reflections
Radiation source: fine-focus sealed tube	1394 reflections with *I* > 2σ(*I*)
graphite	*R*_int_ = 0.047
ω scans	θ_max_ = 26.1°, θ_min_ = 2.3°
Absorption correction: multi-scan (SADABS; Bruker, 2002)	*h* = −8→8
*T*_min_ = 0.978, *T*_max_ = 0.992	*k* = −12→12
7636 measured reflections	*l* = −16→16

### Refinement

**Table:**

Refinement on *F*^2^	Primary atom site location: structure-invariant direct methods
Least-squares matrix: full	Secondary atom site location: difference Fourier map
*R*[*F*^2^ > 2σ(*F*^2^)] = 0.058	Hydrogen site location: inferred from neighbouring sites
*wR*(*F*^2^) = 0.180	H-atom parameters constrained
*S* = 0.93	*w* = 1/[σ^2^(*F*_o_^2^) + (0.0762*P*)^2^] where *P* = (*F*_o_^2^ + 2*F*_c_^2^)/3
3444 reflections	(Δ/σ)_max_ < 0.001
253 parameters	Δρ_max_ = 0.34 e Å^−^^3^
0 restraints	Δρ_min_ = −0.18 e Å^−^^3^

### Special details

**Table:**

Geometry. All e.s.d.'s (except the e.s.d. in the dihedral angle between two l.s. planes) are estimated using the full covariance matrix. The cell e.s.d.'s are taken into account individually in the estimation of e.s.d.'s in distances, angles and torsion angles; correlations between e.s.d.'s in cell parameters are only used when they are defined by crystal symmetry. An approximate (isotropic) treatment of cell e.s.d.'s is used for estimating e.s.d.'s involving l.s. planes.
Refinement. Refinement of *F*^2^ against ALL reflections. The weighted *R*-factor *wR* and goodness of fit *S* are based on *F*^2^, conventional *R*-factors *R* are based on *F*, with *F* set to zero for negative *F*^2^. The threshold expression of *F*^2^ > σ(*F*^2^) is used only for calculating *R*-factors(gt) *etc*. and is not relevant to the choice of reflections for refinement. *R*-factors based on *F*^2^ are statistically about twice as large as those based on *F*, and *R*- factors based on ALL data will be even larger.

### Fractional atomic coordinates and isotropic or equivalent isotropic displacement parameters (Å^2^)

**Table:**

	*x*	*y*	*z*	*U*_iso_*/*U*_eq_	
C1	0.7589 (5)	0.5815 (4)	1.2595 (3)	0.0603 (10)	
H1	0.7594	0.6000	1.3295	0.072*	
C2	0.7631 (5)	0.6947 (4)	1.2121 (3)	0.0593 (9)	
H2	0.7692	0.7861	1.2495	0.071*	
C3	0.7581 (4)	0.6668 (4)	1.1082 (3)	0.0534 (9)	
H3	0.7589	0.7394	1.0739	0.064*	
C4	0.7517 (4)	0.5297 (3)	1.0542 (2)	0.0411 (8)	
C5	0.7492 (4)	0.4942 (3)	0.9448 (2)	0.0418 (8)	
C6	0.7476 (4)	0.5624 (4)	0.7934 (3)	0.0473 (8)	
C7	0.7508 (5)	0.6785 (4)	0.7365 (2)	0.0607 (10)	
H7A	0.8810	0.7585	0.7590	0.073*	
H7B	0.6518	0.7193	0.7543	0.073*	
C8	0.7086 (6)	0.6201 (4)	0.6202 (3)	0.0773 (12)	
H8A	0.5654	0.5694	0.5943	0.093*	
H8B	0.7534	0.7030	0.5898	0.093*	
C9	0.8103 (6)	0.5164 (4)	0.5882 (3)	0.0779 (12)	
H9A	0.9537	0.5678	0.6125	0.093*	
H9B	0.7826	0.4845	0.5137	0.093*	
C10	0.7414 (6)	0.3822 (4)	0.6306 (2)	0.0669 (10)	
H10A	0.6063	0.3186	0.5940	0.080*	
H10B	0.8271	0.3265	0.6187	0.080*	
C11	0.7453 (4)	0.4230 (4)	0.7430 (2)	0.0485 (8)	
C12	0.7473 (4)	0.3561 (3)	0.8947 (2)	0.0433 (8)	
C13	0.7493 (4)	0.2468 (3)	0.9524 (2)	0.0439 (8)	
C14	0.7547 (4)	0.1100 (4)	0.9065 (3)	0.0535 (9)	
H14	0.7545	0.0847	0.8367	0.064*	
C15	0.7602 (5)	0.0130 (4)	0.9645 (3)	0.0578 (9)	
H15	0.7664	−0.0782	0.9355	0.069*	
C16	0.7563 (5)	0.0539 (4)	1.0681 (3)	0.0622 (10)	
H16	0.7582	−0.0131	1.1071	0.075*	
C17	0.7489 (4)	0.2799 (3)	1.0574 (2)	0.0431 (8)	
C18	0.7504 (4)	0.4245 (3)	1.1102 (2)	0.0429 (8)	
C19	0.7010 (6)	0.0878 (4)	1.3406 (3)	0.0611 (10)	
C20	0.5949 (5)	0.0433 (3)	1.4226 (2)	0.0527 (9)	
C21	0.4077 (6)	0.0460 (4)	1.4252 (3)	0.0597 (10)	
H21	0.3439	0.0761	1.3747	0.072*	
C22	0.6860 (5)	−0.0042 (4)	1.4971 (3)	0.0611 (10)	
H22	0.8116	−0.0082	1.4950	0.073*	
N1	0.7543 (4)	0.4499 (3)	1.2122 (2)	0.0533 (7)	
N2	0.7502 (4)	0.1824 (3)	1.1147 (2)	0.0513 (7)	
N3	0.7496 (4)	0.5981 (3)	0.89318 (19)	0.0472 (7)	
N4	0.7442 (4)	0.3203 (3)	0.79299 (19)	0.0491 (7)	
O1	0.8422 (4)	0.0570 (3)	1.3217 (2)	0.0853 (9)	
O2	0.6271 (4)	0.1674 (3)	1.29255 (18)	0.0766 (8)	
H2A	0.6112	0.1355	1.2302	0.115*	

### Atomic displacement parameters (Å^2^)

**Table:**

	*U*^11^	*U*^22^	*U*^33^	*U*^12^	*U*^13^	*U*^23^
C1	0.067 (2)	0.068 (3)	0.049 (2)	0.028 (2)	0.0207 (18)	0.014 (2)
C2	0.070 (2)	0.053 (2)	0.057 (3)	0.0253 (19)	0.0208 (19)	0.0101 (19)
C3	0.054 (2)	0.053 (2)	0.058 (2)	0.0230 (18)	0.0178 (18)	0.0190 (19)
C4	0.0385 (18)	0.0422 (19)	0.043 (2)	0.0169 (15)	0.0081 (15)	0.0082 (16)
C5	0.0338 (18)	0.046 (2)	0.045 (2)	0.0133 (15)	0.0059 (15)	0.0148 (17)
C6	0.0439 (19)	0.050 (2)	0.049 (2)	0.0188 (17)	0.0066 (16)	0.0151 (18)
C7	0.069 (2)	0.065 (2)	0.051 (2)	0.0243 (19)	0.0119 (19)	0.0243 (19)
C8	0.106 (3)	0.082 (3)	0.053 (3)	0.043 (3)	0.015 (2)	0.026 (2)
C9	0.100 (3)	0.093 (3)	0.057 (2)	0.047 (3)	0.025 (2)	0.029 (2)
C10	0.084 (3)	0.070 (3)	0.043 (2)	0.027 (2)	0.0104 (19)	0.0135 (19)
C11	0.049 (2)	0.052 (2)	0.041 (2)	0.0161 (17)	0.0074 (16)	0.0106 (18)
C12	0.0388 (18)	0.047 (2)	0.042 (2)	0.0156 (15)	0.0067 (15)	0.0103 (17)
C13	0.0391 (18)	0.044 (2)	0.048 (2)	0.0137 (15)	0.0108 (15)	0.0134 (17)
C14	0.058 (2)	0.050 (2)	0.052 (2)	0.0230 (18)	0.0093 (17)	0.0091 (19)
C15	0.070 (2)	0.043 (2)	0.064 (2)	0.0256 (19)	0.0160 (19)	0.0128 (19)
C16	0.072 (2)	0.049 (2)	0.067 (3)	0.0211 (19)	0.015 (2)	0.023 (2)
C17	0.0412 (18)	0.0398 (19)	0.049 (2)	0.0132 (15)	0.0120 (15)	0.0146 (17)
C18	0.0401 (18)	0.048 (2)	0.043 (2)	0.0173 (16)	0.0116 (15)	0.0135 (17)
C19	0.079 (3)	0.053 (2)	0.057 (2)	0.025 (2)	0.024 (2)	0.0211 (19)
C20	0.067 (2)	0.043 (2)	0.050 (2)	0.0207 (18)	0.0185 (18)	0.0124 (17)
C21	0.074 (3)	0.059 (2)	0.054 (2)	0.030 (2)	0.0161 (19)	0.0227 (19)
C22	0.069 (2)	0.060 (2)	0.066 (2)	0.0287 (19)	0.023 (2)	0.025 (2)
N1	0.0634 (18)	0.0523 (19)	0.0464 (18)	0.0224 (15)	0.0166 (14)	0.0131 (15)
N2	0.0600 (18)	0.0427 (17)	0.0547 (18)	0.0202 (14)	0.0139 (14)	0.0171 (15)
N3	0.0502 (16)	0.0494 (17)	0.0433 (17)	0.0193 (13)	0.0087 (13)	0.0143 (14)
N4	0.0541 (17)	0.0485 (17)	0.0435 (18)	0.0194 (14)	0.0095 (13)	0.0098 (14)
O1	0.099 (2)	0.097 (2)	0.101 (2)	0.0578 (18)	0.0578 (18)	0.0550 (17)
O2	0.101 (2)	0.0836 (19)	0.0687 (17)	0.0465 (16)	0.0330 (15)	0.0399 (15)

### Geometric parameters (Å, °)

**Table:**

C1—N1	1.323 (4)	C10—H10B	0.9700
C1—C2	1.393 (4)	C11—N4	1.329 (4)
C1—H1	0.9300	C12—N4	1.349 (4)
C2—C3	1.375 (4)	C12—C13	1.461 (4)
C2—H2	0.9300	C13—C14	1.392 (4)
C3—C4	1.393 (4)	C13—C17	1.397 (4)
C3—H3	0.9300	C14—C15	1.366 (4)
C4—C18	1.405 (4)	C14—H14	0.9300
C4—C5	1.452 (4)	C15—C16	1.389 (4)
C5—N3	1.355 (3)	C15—H15	0.9300
C5—C12	1.398 (4)	C16—N2	1.322 (4)
C6—N3	1.327 (4)	C16—H16	0.9300
C6—C11	1.408 (4)	C17—N2	1.357 (4)
C6—C7	1.504 (4)	C17—C18	1.466 (4)
C7—C8	1.520 (4)	C18—N1	1.351 (4)
C7—H7A	0.9700	C19—O1	1.208 (4)
C7—H7B	0.9700	C19—O2	1.317 (4)
C8—C9	1.486 (5)	C19—C20	1.488 (5)
C8—H8A	0.9700	C20—C21	1.375 (4)
C8—H8B	0.9700	C20—C22	1.381 (4)
C9—C10	1.511 (4)	C21—C22^i^	1.384 (4)
C9—H9A	0.9700	C21—H21	0.9300
C9—H9B	0.9700	C22—C21^i^	1.384 (4)
C10—C11	1.491 (4)	C22—H22	0.9300
C10—H10A	0.9700	O2—H2A	0.8200
			
N1—C1—C2	124.8 (3)	N4—C11—C6	121.7 (3)
N1—C1—H1	117.6	N4—C11—C10	116.5 (3)
C2—C1—H1	117.6	C6—C11—C10	121.8 (3)
C3—C2—C1	117.8 (3)	N4—C12—C5	121.5 (3)
C3—C2—H2	121.1	N4—C12—C13	118.5 (3)
C1—C2—H2	121.1	C5—C12—C13	120.1 (3)
C2—C3—C4	119.9 (3)	C14—C13—C17	118.3 (3)
C2—C3—H3	120.1	C14—C13—C12	122.0 (3)
C4—C3—H3	120.1	C17—C13—C12	119.7 (3)
C3—C4—C18	117.4 (3)	C15—C14—C13	119.6 (3)
C3—C4—C5	122.5 (3)	C15—C14—H14	120.2
C18—C4—C5	120.1 (3)	C13—C14—H14	120.2
N3—C5—C12	121.2 (3)	C14—C15—C16	118.3 (3)
N3—C5—C4	118.5 (3)	C14—C15—H15	120.8
C12—C5—C4	120.3 (3)	C16—C15—H15	120.8
N3—C6—C11	121.7 (3)	N2—C16—C15	124.0 (3)
N3—C6—C7	116.9 (3)	N2—C16—H16	118.0
C11—C6—C7	121.4 (3)	C15—C16—H16	118.0
C6—C7—C8	113.5 (3)	N2—C17—C13	122.2 (3)
C6—C7—H7A	108.9	N2—C17—C18	117.4 (3)
C8—C7—H7A	108.9	C13—C17—C18	120.4 (3)
C6—C7—H7B	108.9	N1—C18—C4	123.4 (3)
C8—C7—H7B	108.9	N1—C18—C17	117.1 (3)
H7A—C7—H7B	107.7	C4—C18—C17	119.4 (3)
C9—C8—C7	112.1 (3)	O1—C19—O2	123.7 (4)
C9—C8—H8A	109.2	O1—C19—C20	123.6 (4)
C7—C8—H8A	109.2	O2—C19—C20	112.6 (4)
C9—C8—H8B	109.2	C21—C20—C22	119.2 (3)
C7—C8—H8B	109.2	C21—C20—C19	121.8 (3)
H8A—C8—H8B	107.9	C22—C20—C19	119.0 (4)
C8—C9—C10	111.6 (3)	C20—C21—C22^i^	120.1 (3)
C8—C9—H9A	109.3	C20—C21—H21	119.9
C10—C9—H9A	109.3	C22^i^—C21—H21	119.9
C8—C9—H9B	109.3	C20—C22—C21^i^	120.6 (3)
C10—C9—H9B	109.3	C20—C22—H22	119.7
H9A—C9—H9B	108.0	C21^i^—C22—H22	119.7
C11—C10—C9	112.4 (3)	C1—N1—C18	116.7 (3)
C11—C10—H10A	109.1	C16—N2—C17	117.6 (3)
C9—C10—H10A	109.1	C6—N3—C5	117.0 (3)
C11—C10—H10B	109.1	C11—N4—C12	116.9 (3)
C9—C10—H10B	109.1	C19—O2—H2A	109.5
H10A—C10—H10B	107.9		

Symmetry codes: (i) −*x*+1, −*y*, −*z*+3.

### Hydrogen-bond geometry (Å, °)

**Table:**

*D*—H···*A*	*D*—H	H···*A*	*D*···*A*	*D*—H···*A*
O2—H2A···N2	0.82	2.02	2.694 (4)	139
